# Microstructure and mechanics of human resistance arteries

**DOI:** 10.1152/ajpheart.00002.2016

**Published:** 2016-09-23

**Authors:** J. S. Bell, A. O. Adio, A. Pitt, L. Hayman, C. E. Thorn, A. C. Shore, J. L. Whatmore, C. P. Winlove

**Affiliations:** ^1^Department of Physics, University of Exeter, Exeter, United Kingdom;; ^2^Diabetes and Vascular Medicine, Institute of Biomedical and Clinical Sciences, University of Exeter Medical School and NIHR Exeter Clinical Research Facility, Exeter, United Kingdom; and; ^3^Institute of Biomedical and Clinical Science, University of Exeter Medical School, University of Exeter, Exeter, United Kingdom

**Keywords:** resistance artery, blood pressure, extracellular matrix, stress, mechanical modeling

## Abstract

Vascular diseases such as diabetes and hypertension cause changes to the vasculature that can lead to vessel stiffening and the loss of vasoactivity. The microstructural bases of these changes are not presently fully understood. We present a new methodology for stain-free visualization, at a microscopic scale, of the morphology of the main passive components of the walls of unfixed resistance arteries and their response to changes in transmural pressure. Human resistance arteries were dissected from subcutaneous fat biopsies, mounted on a perfusion myograph, and imaged at varying transmural pressures using a multimodal nonlinear microscope. High-resolution three-dimensional images of elastic fibers, collagen, and cell nuclei were constructed. The honeycomb structure of the elastic fibers comprising the internal elastic layer became visible at a transmural pressure of 30 mmHg. The adventitia, comprising wavy collagen fibers punctuated by straight elastic fibers, thinned under pressure as the collagen network straightened and pulled taut. Quantitative measurements of fiber orientation were made as a function of pressure. A multilayer analytical model was used to calculate the stiffness and stress in each layer. The adventitia was calculated to be up to 10 times as stiff as the media and experienced up to 8 times the stress, depending on lumen diameter. This work reveals that pressure-induced reorganization of fibrous proteins gives rise to very high local strain fields and highlights the unique mechanical roles of both fibrous networks. It thereby provides a basis for understanding the micromechanical significance of structural changes that occur with age and disease.

## Abstract (precis)

*This is the first study to elucidate and quantify the microstructural bases of the mechanical properties of human resistance arteries. The geometrically accurate mechanical analysis provides new insights into strain fields existing in the walls of small arteries, and raises questions about the mechanobiology of vascular remodeling*.

## NEW & NOTEWORTHY

*This is the first study to elucidate and quantify the microstructural bases of the mechanical properties of human resistance arteries. The geometrically accurate mechanical analysis provides new insights into strain fields existing in the walls of small arteries, and raises questions about the mechanobiology of vascular remodeling*.

the network of small resistance arteries and arterioles is the main contributor to vascular resistance and, through the arteries' ability actively to change their diameter, an important regulator of local tissue perfusion. However, the passive and active mechanical properties of these vessels have generally been characterized only in terms of gross changes in vessel radius or wall thickness/area in response to changes in transmural pressure or smooth muscle tone. This scale of characterization is inadequate to understand processes such as the transmission of mechanical signals between blood and vascular cells and the functional significance of the structural and cellular changes that occur with age and in many diseases.

The mechanical properties of large blood vessels are largely passive, and the relationship between their nonlinear stress-strain behavior and the extensive networks of compliant elastic fibers and much more rigid collagen fibers has been revealed over many years (13, 55). In small blood vessels smooth muscle cells make an active contribution to vascular mechanics, and the ways in which smooth muscle tone is determined both by chemical signals and by the complex patterns of mechanical forces, including fluid- and solid-shear stress and pressure, have been extensively documented (21, 35). However, our understanding of small vessel biomechanics is otherwise still somewhat limited. Small artery mechanics are generally characterized by a singular “stiffness,” a parameter that is used, for example, to characterize vessel remodeling in disease (38, 39, 56). Stiffness is generally determined from microscopic measurements of the apparent internal and external diameters of a vessel mounted on a pressure myograph (18, 30), and analysis is based on the assumption that the blood vessel is homogeneous in its composition and mechanical properties. The vast differences in the mechanical properties of different regions of blood vessels, which has been demonstrated in large arteries by, for example, the work of Holzapfel et al. (24, 25), show that whole vessel calculations of quantities such as wall stress and radial strain do not reflect the intramural mechanical environment. There is an urgent need to extend microscale analysis to the small vessels.

The manner in which an elastic fiber network allows large-scale distention, which is arrested by a network of collagen fibers that prevent damaging overextension, has been observed in many tissues (16, 40). In large arteries significant progress has been made in quantifying the orientation of fibrous networks (44), the mechanical relationship between collagen and elastic fibers (10, 15, 50), and the mechanical effect of vascular tone (54). To date there has been no similar characterization of small arteries. The radial distension of small arteries under increasing transmural pressure in the absence of myogenic responses has been described by multiparameter “hook-on” models (3) and, more recently, serial element models (52). These are essentially two-phase linear or hyperelastic models that account for strain-dependent recruitment of collagen fibers and urgently require support from microstructural observations.

To test and extend models to a level where they can be used to evaluate the functional significance of changes in structure and composition, it is necessary not only to visualize the three-dimensional structure of the intact vessel, but also continuously to observe the changes that occur during pressurization or alterations in muscle tone. Only against this background can the functional significance of changes that occur with age (12), lifestyle (18), disease (34), and body location (11) be fully understood. These objectives can be realized using nonlinear microscopy (NLM), a technique that recently has been reviewed in the context of vascular disease (32) and employed in the characterization of static resistance arteries (6).

In the present investigation, unfixed human small resistance arteries mounted in a perfusion myograph were repeatedly imaged as transmural pressure was increased. Two-photon fluorescence (TPF) revealed elastic fibers and other intracellular autofluorescent proteins while second-harmonic generation (SHG) revealed fibrous collagen. Three-dimensional reconstructions of the coregistered TPF and SHG images revealed separate layers in the extracellular matrix of the vessel wall, while nuclear staining using 4′,6-diamidino-2-phenylindole dilactate (DAPI) provided information about the distribution of cells in relation to these layers. A simple two-layer analytical model of the vessel wall was constructed using the images to determine the distribution of strain and thereby to infer the variation in stiffness and circumferential stress across the vessel wall. The mechanical interplay between the highly extensible elastic fiber network and the inextensible collagen network is analyzed by calculating the distributions of orientation, and changes associated with increased transmural pressure.

## MATERIALS AND METHODS

The study was performed on resistance arteries from healthy human volunteers recruited from the Exeter Ten Thousand cohort. Following the administration of a local anesthetic a subcutaneous abdominal adipose tissue biopsy was removed by scalpel incision at 10 cm laterally to the right of the umbilicus and immediately transported to the microscopy laboratory. Fully informed written consent was obtained in accordance with the Declaration of Helsinki. Ethics approval was granted by the NRES Committee South West, Exeter (Ref. no.: 11/SW/0199).

During transport and dissection, the tissue sample was immersed in 3-[*N*-morpholino]propane sulfonic acid (MOPS) buffer (at 4°C) containing (in mmol/l): 145 NaCl, 4.7 KCl, 2.0 CaCl_2_(2H_2_O) 1.17 MgSO_4_(7H_2_O), 2.0 MOPS, 1.2 NaH_2_PO_4_(H_2_O), 5.0 glucose, 2.0 pyruvate, 0.02 EDTA, and 2.75 NaOH adjusted to pH 7.40 ± 0.02. Small resistance arteries were visualized under a dissection microscope, and adipocytes and excess connective tissue were removed leaving the adventitia intact. Segments ∼3–5 mm in length with an outer diameter of 200–400 μm and without visible side branches were deemed suitable for cannulation. Arteries were carefully transferred to a custom-made myograph bath containing chilled MOPS buffer (4°C).

The vessel preparation protocol was adapted from a previous study (28). The dissected resistance arteries were cannulated with glass capillary tubes pulled to a diameter of ∼20 μm attached to the myograph and secured with 11-0 gauge suture (Ethicon). During the mounting process the vessels were perfused to remove blood from the lumen, taking care to prevent introducing bubbles and damage to the endothelium. The capillary tubes were then moved apart until the vessels were straight but not stretched before being placed on the microscope stage. The myograph bath was maintained at 37°C using a pump and heat exchanger. Transmural pressure was controlled using medium-filled pressure reservoirs connected to the capillary tubes, which were maintained at a minimum transmural pressure of 3 mmHg to prevent vessel collapse. Images were acquired at 3, 10, 20, and 30 mm and 50 mmHg (the physiological pressure range in these vessels is believed to be 40–90 mmHg, as discussed below). Vessels were allowed to equilibrate for 15 min following a pressure increment before imaging. The myograph included the facility to adjust longitudinal strain if vessels bent following changes in pressure, although it was not needed.

The nonlinear microscopy system comprised a modified confocal laser-scanning microscope (FluoView IX71 and F300; Olympus) and Ti-sapphire laser (816 nm, Mira 900-D; Coherent) pumped by a 532-nm solid state laser (Verdi V10; Coherent) with a repetition rate of 76 MHz and a pulse width of 100 fs. TPF and SHG signals were separated from the laser fundamental using a long pass dichroic mirror (670dcxr, part 7 in 2.5b; Chroma Technologies) before being separated from one another by a second long pass dichroic mirror (Di02-R405; Semrock). The TPF signal was passed through two band-pass filters (F70-500-3-PFU and CG-BG-39; CVI Melles Griot) centered at 500 nm with full-width half-maximum (FWHM) of 70 nm, and the SHG signal was passed through two band pass filters [FF01-405 (Semrock) and CG-BG-39 (CVI Melles Griot)] centered at 405 nm with FWHM of 10 nm. This matches closely the spectral peak of TPF for elastin (36) and that of SHG for collagen (31). The signal was focused into a photomultiplier tube (Hamamatsu R3896). Olympus UPlanSApo ×20 0.4 NA and ×60 1 NA water immersion objectives were used to obtain 500 nm resolution en face and sagittal images to a depth of up to 200 μm. Each 512 × 512 pixel image took 22 s to capture and was incremented in *z* by 1 μm. A typical 135-μm stack therefore took ∼50 min to complete. After the 30 mmHg SHG/TPF imaging protocol, for vessels that were not chosen for the incremental layer stress study, DAPI nuclear stain (Sigma) was mixed in the bathing solution at a final concentration of 500 nM. An image stack was then taken to reveal the distribution of cell nuclei.

Image stacks were converted into three-dimensional images using the Volume Viewer plugin for Fiji (42). Vessel radii and wall layer thicknesses were calculated by fitting circles to the inner and outer vessel boundaries, as well as the interface between the media and adventitia, which was demarcated by a step in SHG signal. Where vessels had irregular shapes, circles were fitted such that circle area matched that of the region of interest. Measurements were taken for at least five longitudinal points per vessel and verified by plotting signal intensity profiles through the wall. The OrientationJ Distribution plugin for Fiji (37) was used to quantify the orientation of the intimal and adventitial elastic fiber networks, and the collagen network.

The wall strains associated with increasing luminal pressure were modeled analytically using linear thick-walled cylinder theory (see Ref. 53 for a full derivation). Briefly, assuming negligible torsion and axial strain, the displacement field u = u_*r*_ for each layer is described by the continuity equation:

(1) $$\frac{{\text{d}}^{2}{\text{u}}_{r}}{\text{d}r^{2}}+\frac{1}{r}\frac{{\text{du}}_{r}}{\text{d}r}-\frac{{\text{u}}_{r}}{r^{2}}=0$$

with the general solution:

(2) $${\text{u}}_{r}=\frac{{\text{C}}_{r1}}{r}+{\text{C}}_{r2}r$$

Assuming radial displacement and stresses are continuous across the layer interface and that the pressure outside the vessel is zero, the values of C_*r1*_ and C_*r2*_ can be calculated for each layer from the inner radius *r*_i_, the outer radius *r*_o_, the elastic modulus *E*, the Poisson's ratio ν, and the lumen pressure p. Radial deformations were calculated for given *E*_i_, ν_i_, *E*_o_, and ν_o_ (subscripts i and o refer to inner and outer layer, respectively), and a brute-force minimization algorithm was written to find the material parameters that optimally map vessel geometry at 3 mmHg transmural pressure to that at 30 mmHg. The objective of the algorithm was to minimize the sum of the squares of the errors in the layer boundary positions. The same model was used to determine material parameters for a homogeneous model assuming the vessels to be a single layer.

Standard hoop stress, σ, referred to in myography as “media stress” (27), in a homogeneous tube is defined as:

(3) $$\sigma =\frac{\text{p}r_{i}}{r_{\text{o}}^{2}-r_{\text{i}}^{2}}+\frac{\text{p}r_{\text{i}}^{2}r_{\text{o}}^{2}}{r^{2}\left(r_{\text{o}}^{2}-r_{\text{i}}^{2}\right)}$$

Circumferential stress in the two-layer model is defined as

(4) $$\sigma_{\theta \theta }=\text{E}\frac{\left(1-2\text{ν}\right){\text{C}}_{r1}+r^{2}{\text{C}}_{r2}}{r^{2}\left(1-2\text{ν}\right)\left(1+\text{ν}\right)}$$

and, to act as a true comparator to hoop stress, the effect of circumferential expansion pressure due to radial strain is ignored.

Unless otherwise stated, data are presented as means ± SE. Statistical significance was calculated using *t*-tests with the null hypothesis rejected at the 5% significance level. The Spearman correlation was used to determine whether changes in features on orientation plots correlated with changes in mechanical properties.

## RESULTS

Vessels were obtained from 12 healthy subjects. Table 1 summarises the measurements taken from each volunteer/sample. It was possible to discern some commonly occurring structural features and responses to increasing pressures, which we describe first. We then describe marked differences that were observed in some vessels.

**Table 1.** Statistical summary of the gender, BMI, age, and BP of the volunteers in this study and the dimensions of the resistance arteries obtained from subcutaneous fat biopsies at 30 mmHg transmural pressure

Subject and Sample Summary	
Gender, M/F	8/4
BMI	24.9 ± 2.5*
Age, yr	53.6 ± 9.2*
Systolic BP, mmHg	122.8 ± 10.1*
Diastolic BP, mmHg	75.4 ± 8.1*
Inner radius, μm	117 ± 17
Wall-to-lumen ratio	0.37 ± 0.07
Adventitia-to-media ratio	0.82 ± 0.12

Values are means ± SE unless otherwise indicated. M, males; F, females; BMI, body mass index; BP, blood pressure.

* Means ± SD.

### 

#### Vessel morphology.

TPF imaging showed two morphologically distinct networks of elastic fibers, one that formed an internal elastic layer (IEL) and another in the adventitia, whose density varied between subjects. The IEL contained longitudinally aligned fibers up to 5 μm in diameter, braced by thinner fibers <1–2 μm in diameter. Adventitial elastic fibers were generally <1 μm in diameter, although fibers up to 3 μm in diameter were sometimes found, particularly in vessels with denser adventitial elastic fiber networks. SHG revealed a network of collagen fibers forming bundles between 3 and 35 μm in diameter in the adventitia. No fibrous collagen was found in the media or intima of any subjects.

**Fig. 1.**
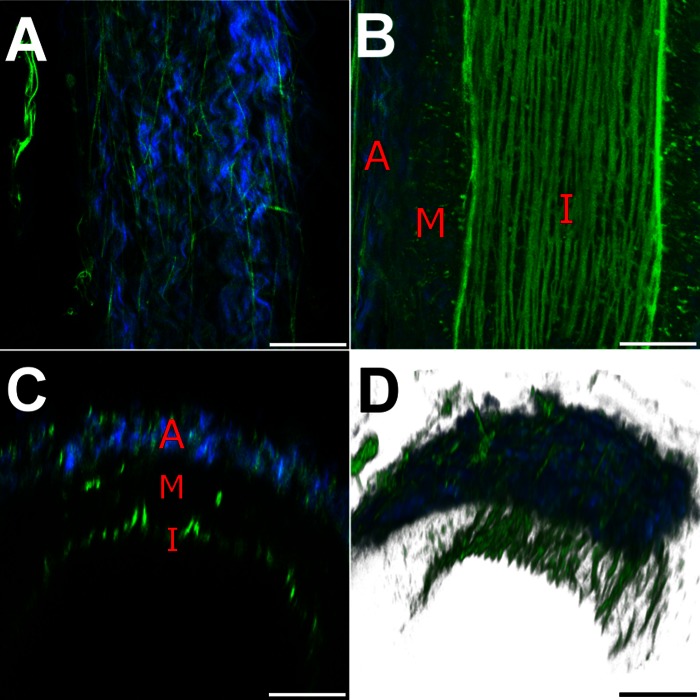
False color images of a 138-μm-lumen-diameter vessel at 3 mmHg transmural pressure showing second-harmonic generation (SHG, collagen) in blue and two-photon fluorescence (TPF, elastic fibers and cellular fluorescence) in green. Red labels: adventitia (A), media (M), and intima (I). *A*: optical section through the adventitia showing wavy collagen punctuated by thin elastic fibers. *B*: reconstruction of a section along the central vessel axis showing thick longitudinally aligned elastic fibers of the internal elastic layer (IEL). *C*: axial section. *D*: 3-dimensional (3D) section of the imaged region of the vessel. Bars, 50 μm.

**Fig. 2.**
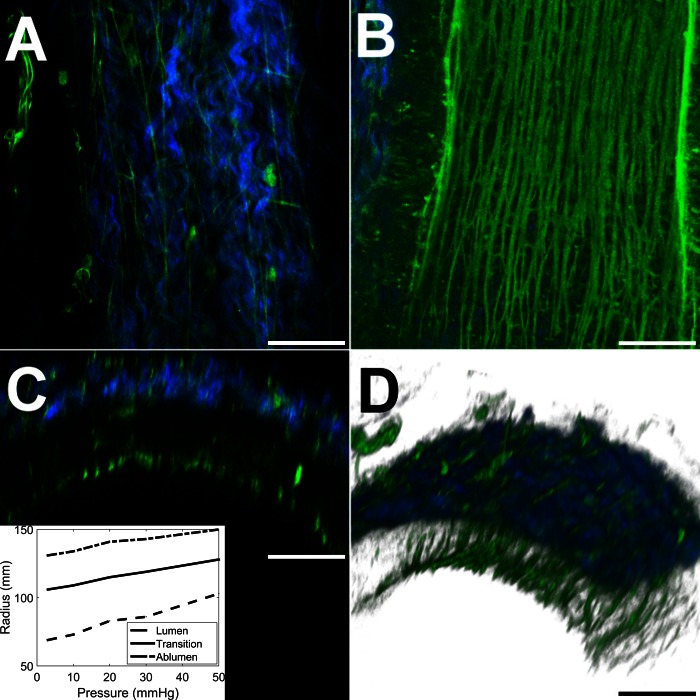
False color images of the vessel pictured in Fig. 1 raised to a transmural pressure of 30 mmHg causing the lumen to dilate to a diameter of 172 μm. *A*: optical section through the adventitia showing a visually relatively unchanged fibrous matrix. *B*: reconstruction of a section along the central vessel axis. Gaps caused by intimal dilation appear between the elastic fibers of the IEL, which in places bulge apart, braced by thinner connecting fibers. *C*: axial section. *D*: 3D section of the imaged region of the vessel. Bars, 50 μm.

Figures 1 and 2 show coregistered TPF (green, predominantly elastic fibers) and SHG (blue, collagen) images of a typical vessel obtained from *subject 11* at transmural pressures of 3 and 30 mmHg, respectively. Supplemental videos S1, *A*–*D*, show progressive sections through the artery in each of the imaging modalities (Supplemental data for this article can be found on the Journal website.).

At the lower pressure the average internal diameter was 138 μm and the average external diameter 262 μm, giving an average wall thickness of 62 μm. Figure 1*A* shows a longitudinal section through the adventitia at low pressure, which comprises discrete bundles of wavy or helically wound collagen, which are interwoven and punctuated by a sparse network of adventitial elastic fibers. Both components are predominantly aligned longitudinally. The collagen bundles have a helical/wavy periodicity of between 20 and 50 μm, scaling with bundle diameter. Elastic fibers run between and through the collagen helices and pass continuously across the adventitia-media boundary. Figure 1*B* shows a longitudinal section through the vessel wall with the adventitia and media at the sides and the IEL in the middle. Longitudinal fibers of the IEL appear closely packed and occasionally overlap, with bracing fibers spaced at regular intervals of 15–20 μm. The small spots of TPF in the media are believed to be fluorescent cellular proteins. Figure 1, *C* and *D*, shows radial sections and a three-dimensional perspective view of the vessel, respectively. These views reveal the fibrous adventitia, the dark predominantly cellular media, and the highly fluorescent IEL of the intima. The adventitia is 25 μm thick while the intima and media combined form a layer 37 μm thick.

Raising the transmural pressure to 30 mmHg causes the internal and external diameters to increase to 172 and 286 μm, respectively. The corresponding average circumferential strains at the inner and outer edges of the vessel are 25 and 9%, respectively, while the vessel wall volumetric strain is 5%. The adventitia, pictured in Fig. 2*A*, exhibits slightly straighter collagen fiber bundles, oriented less predominantly in the longitudinal direction. The IEL, pictured in Fig. 2*B*, accommodates the high lumen strain by increasing the gaps between the longitudinal fibers, with the thinner bracing fibers forming a honeycomb structure in places. This pattern of deformation results in a very heterogeneous distribution of local strains, peaking at over 200% in the region between fibers. The section and three-dimensional views in Fig. 2, *C* and *D*, show that the elastic inner wall assumes a more uniform cylindrical contour as pressure is increased. The radial thickness of the intima and media is reduced by 4 μm to 33 μm while the adventitia reduced by 1 μm to 24 μm. The corresponding radial and volumetric strains for the media are −11 and 4% and for the adventitia are −4 and 19%.

**Fig. 3.**
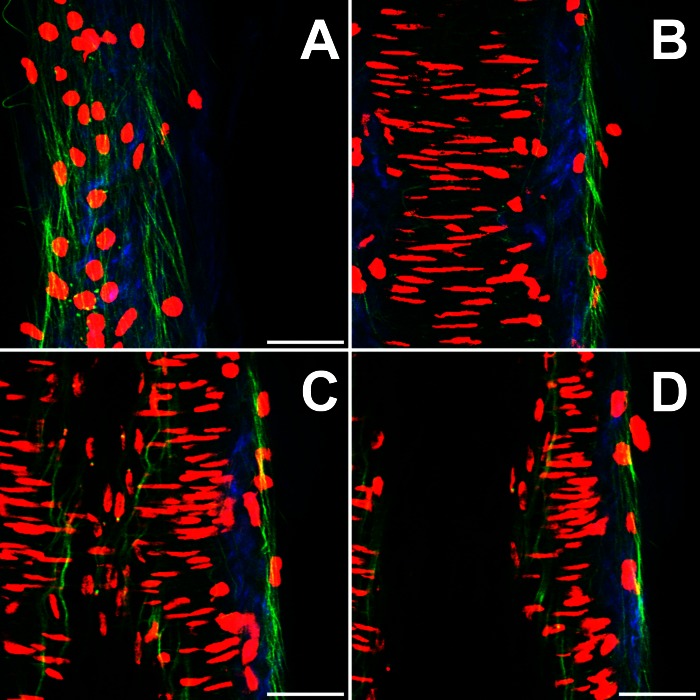
False color images of the distribution of elastin (green), collagen (blue), and cell nuclei stained with 4′,6-diamidino-2-phenylindole dilactate (DAPI, red) of a vessel at 30 mmHg transmural pressure with a 268-μm lumen diameter. Yellow labels: adventitia, media, and intima. *A*: section through the adventitia showing adventitial textured collagen and cell nuclei. *B*: section through the adventitia and media showing slender vascular smooth muscle cell (VSMC) nuclei. *C*: section through the adventitia, media, and intima showing the IEL and longitudinally aligned endothelial nuclei. *D*: section through the wall and lumen. Bars, 50 μm.

Figure 3 shows sections through a vessel obtained from *subject 9*, 332 μm in outer diameter at 30 mmHg transmural pressure, stained with DAPI for cell nucleus visualization. Figure 3, *A*, *B*, *C*, *D*, are taken at positions of 10, 47, 68, and 86 μm, respectively, from the outer edge. Adventitial cells (presumed to be fibroblasts) exhibit no preferential orientation, medial vascular smooth muscle cells (VSMCs) are predominantly aligned circumferentially, and intimal endothelial cells are aligned longitudinally. The positions of endothelial cell nuclei bear no spatial relationship relative to individual fibers in the IEL: nuclei are observed in positions adjacent to elastic fiber intersections, as well as midway between. The radial distance between endothelial cell nuclei and the IEL is below the resolution of the microscope, meaning the basement membrane, which contains type IV collagen and does not generate SHG, must occupy a region <1 μm thick. The VSMC nuclei have a high slenderness ratio and are aligned circumferentially, with a slight helical bias. They vary in length between 17 and 44 μm, with an average of 31 μm. The muscle cell nuclei occupy 20 ± 1% of the medial volume in all vessels. The innermost layer of VSMCs presses against the IEL. The outer VSMC nuclei and adventitial collagen border, and in some cases slightly interpenetrate, one another.

#### Individual variations.

**Fig. 4.**
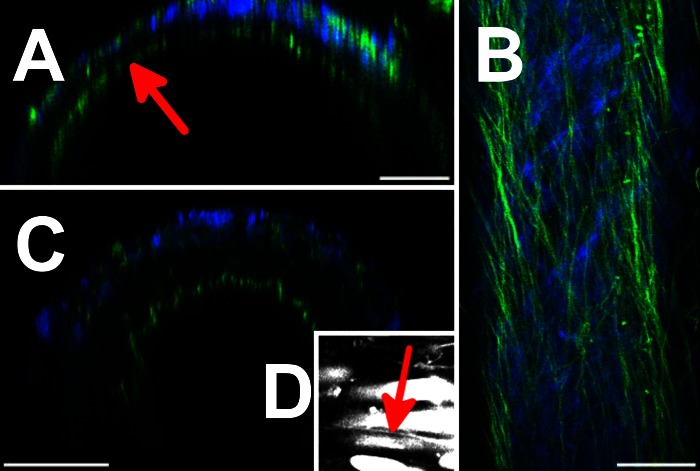
Variations in morphology of vessels. *A*: axial optical section of a large vessel exhibiting wall thinning (arrow). *B*: optical section of adventitia of an atypical vessel with significantly increased elastic fiber content. *C*: axial optical section through a vessel undergoing myogenic contraction, exhibiting a crinkled IEL. *D*: high-zoom TPF image of an elastin fiber located between two VSMCs. Bars = 50 (*A*–*C*) and 25 (*D*) μm.

Five of the 12 vessels were irregular in wall thickness around their circumference, being up to one-third thinner over 2.5–6% of the circumference due to a dip in the outer radius as shown in Fig. 4*A*. The circumferential position of the thin region spiralled around the vessel along its length with an axial periodicity of between 400 and 968 μm.

Three of the 12 vessels showed significantly greater amounts of fibrous protein in the adventitia, such as the example shown in the longitudinal section in Fig. 4*B* and Supplemental Video 2, *A*–*D*. In these vessels the adventitia comprised over one-half the thickness of the vessel wall compared with an overall average of 40%. Adventitial elastic fibers were thicker and formed a continuous layer around the outside of the adventitia up to three fibers thick, whereas the collagen was arranged in thicker more tightly woven bundles. This morphology was distinct from that of the adipose tissue in which the vessel had been embedded. At 30 mmHg transmural pressure the innermost collagen was arranged in straight bundles at ± 45° to the longitudinal direction.

Two vessels underwent myogenic contraction during observation, and in this case the relatively uniform cylindrical morphology of the IEL became corrugated as shown in Fig. 4*C*. These corrugations were up to 5 μm deep and 100 μm long.

Fine elastic fibers <1 μm thick were observed penetrating radially in the media from the adventitia before aligning circumferentially between VSMCs (Fig. 4*D*). These fibers are particularly clear in the TPF supplemental videos.

#### Wall mechanics.

**Fig. 5.**
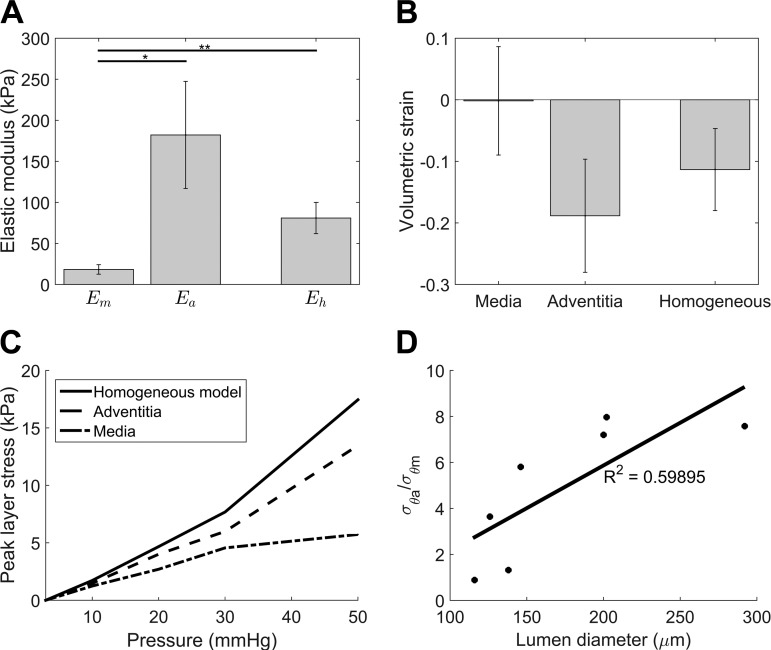
Mechanics data. *A*: elastic moduli assuming a layered vessel wall [media modulus (*E*_m_), adventitia stiffness (*E*_a_)] and a homogeneous wall (*E*_h_). Statistical significance: *P < 0.05 and **P < 0.01. *B*: volumetric strains in the vessel walls. *C*: peak circumferential stress in each layer analyzed for the vessel depicted in Figs. 1 and 2, with increasing transmural pressure. *D*: ratio of adventital circumferential stress (σ_θa_) to medial circumferential stress (σ_θm_) at 30 mmHg transmural pressure with increasing lumen diameter. The positive trend indicates that the adventitia experiences proportionally greater wall stress in larger vessels.

Seven vessels were analyzed using the analytical models. Exclusion criteria included unclear layer boundaries in the images due to scattering, and myogenic events. The geometry and fitted mechanical parameters for the one- and two-layer models are summarized in Table 2 and shown graphically in Fig. 5*A*. In the two-layer model the radial strains in each layer are not significantly different, but the corresponding elastic moduli are (*E*_m_ = 18.2 ± 5.4 kPa vs. *E*_a_ = 182 ± 60 kPa, *P* < 0.05). The range in volumetric strain of both layers (Fig. 5*B*) was considerable, and for the number of vessels examined no significant difference between the layers was established.

**Table 2.** Measured thicknesses of intima and media at low and high transmural pressure and calculated mechanical parameters for the layered and homogeneous models

Geometrical and Mechanical Results				
Layer	Pressure, mmHg	Thickness, μm	Average radial strain, %	Elastic modulus, kPa
Media	3	23.8 ± 3.6	−24 ± 7	18.3 ± 5.4
	30	17.5 ± 2.5		
Adventitia	3	23.0 ± 2.7	−33 ± 7	182 ± 60
	30	14.3 ± 1.2		
Whole wall	3	51.1 ± 4.2	−24 ± 6	81.0 ± 12.9
	30	37.8 ± 3.0		

Values are means ± SE.

An analysis of peak wall stress associated with increasing lumen pressure (referred to as media stress in the myography literature) for the vessel depicted in Figs. 1 and 2 is shown in Fig. 5*C*. At pressures of 30 mmHg and below, the stress is relatively uniform across the vessel, but at 50 mmHg the adventitia experiences more than two times the stress in the media. Over the whole group of vessels the pressure at which the adventitia takes up the majority of the wall stress decreased as the lumen diameter increased. The ratio of adventitial circumferential stress (σ_θa_) to media circumferential stress (σ_θm_) at 30 mmHg is plotted in Fig. 5*D*.

The single layer model, which is widely used to estimate wall stiffness, yielded stiffness values generally between those of the adventitia and media in the two-layer model (*E*_h_ = 67.9 ± 12.9 kPa). This value was significantly different from the media stiffness in the two-layer model (*P* < 0.01). Circumferential stress derived from the single-layer model was generally greater than that calculated directly for the adventitia in the layered model for smaller vessels, but smaller for the larger vessels, and was always many times greater than that in the media. The discrepancies between the two models increased with lumen diameter.

#### Fiber orientation.

**Fig. 6.**
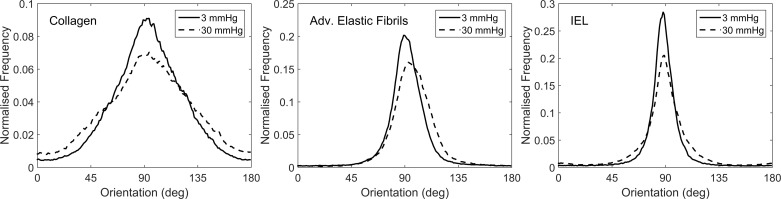
Orientation distributions of the three fibrous networks.

The average spreads of orientation in the fibrous protein networks are shown in Fig. 6, where 90° represents longitudinal alignment and 0°/180° represent circumferential. In all vessels the distributions became more isotropic with distension. The preferred orientation of adventitial elastic fibers moved in the same direction in all samples, suggesting recruitment in a left-handed helical arrangement. Measurements of FWHM (a measure of orientation dispersion), peak orientation, and maximum-to-minimum orientation ratio (a measure of isotropy) were made for each sample at 3 and 30 mmHg transmural pressure and are shown in Table 3.

**Table 3.** Summary of orientation analysis statistics for the three distinct fibrous networks at two pressure increments

	3 mmHg			30 mmHg		
	AEF	IEL	Coll	AEF	IEL	Coll
Peak position, deg	91.3 ± 2.08	88.0 ± 1.1	95.3 ± 4.1	96.6 ± 3.2	89.5 ± 2.0	94.9 ± 4.6
Full width Half max, deg	21.8 ± 3.1	15.7 ± 1.9	62.8 ± 10.5	25.5 ± 2.6	29.7 ± 7.3	82.8 ± 14.3
Min-to-max ratio, AU	2,385 ± 1,304	571 ± 446	172 ± 154	2,401 ± 2,173	205 ± 115	130 ± 121

Values are means ± SE. AEF, adventitial elastic fibers; IEL, internal elastic layer; Coll, fibrous collagen; Min, minimum; Max, maximum; AU, arbitrary units.

There were Spearman correlations (*r*^2^ < 0.05) between adventitial elastic fiber FWHM and adventitia stiffness, adventitial elastic fiber FWHM and adventitia strain, and IEL FWHM and media strain. The ratio of minimum to maximum collagen orientation correlated weakly with both adventitia stiffness and strain (*r*^2^ < 0.1).

## DISCUSSION

The microstructure of human resistance arteries, and changes associated with transmural pressure, have been visualized using NLM and analyzed from a morphological and mechanical perspective. The structure of the small arteries employed in these studies using nonlinear microscopy was consistent with that observed in fixed human pericardial resistance arteries (6), with the exception that in this study no fibrous collagen was observed in the media. The structure of the two fibrous networks in the adventitia was similar to that seen in both large (10, 50) and small (6) arteries. It was notable that none of the vessels contained the fenestrated sheets of elastin reported in the intima of resistance arteries in other studies (11).

A focus of the present research was the structural changes accompanying increases in luminal pressure, which differed in each of the fibrous protein networks. In the IEL the fibers were predominantly axially aligned at low pressure but at higher pressure spread apart to reveal gaps bridged by regularly spaced bracing fibers, which appear on the orientation plot as an emerging peak around 0°. This honeycomb-like structure has been visualized at much higher pressures (6) and its mechanical properties analyzed in large arteries (10).

Collagen fibers were found in this study to straighten with increasing pressure and become more isotropic in orientation, which is a common observation in arteries (10, 15, 50). In some vessels there was radius-dependent recruitment of collagen (see Supplemental Video 2*D*), as has been previously noted in larger arteries (10) with the same pattern of orientation (24). This behavior may be responsible for the reduced distensibility known to be associated with increased transmural pressure (47).

The adventitial elastic fibers exhibited the most unexpected response to transmural pressure in that the preferential orientation shifted to a left-handed helical state. The reorientation was less than that of the neighboring collagen network, demonstrating the complexity of the strain fields at the fibrillar level. We suggest that the fine circumferential elastic fibers in the media act as “anchoring points” and possess a role in restoring the geometry of the networks after cellular relaxation or in distributing interfacial stresses arising from the different mechanical properties of the media and adventitia. Elastin-deficient mice have twisted and tortuous aortas (9), so it is also possible that adventitial elastic fibers mediate torsion in resistance arteries.

The very large local strains between elastic fibers of the IEL raise questions concerning the coupling of VSMCs with matrix fibers. It is known, for example, that displacement by as little as 30 nm of a focal adhesion in a VSMC can provoke a myogenic response (46), and it may be that it is possible that the bracing fibers, which experience far less circumferential strain, act as strain-protected anchoring sites for VSMCs. There are similar questions concerning the attachment of the endothelial cells to the underlying matrix, and a particular issue here is the corrugations that were produced in the IEL during myogenic contraction. The basement membrane cannot be imaged directly (type IV collagen does not produce SHG), but it must be <1 μm in thickness, since no gap between endothelial cell nuclei and elastic fibers could be resolved. Furthermore, endothelial cell nuclei were found both in the grooves of the corrugations and exposed on the ridges, indicating that the cells followed the underlying contours, suggesting an intimate coupling of IEL to basement membrane. Given the local strains between elastic fibers of the IEL were found to exceed 200%, this further suggests that the type IV collagen network that comprises the skeleton of basement membrane structure routinely experiences strains of a similar magnitude. Whereas type IV collagen is believed to be quite stiff, network arrangements such as honeycomb or chicken wire have been proposed (51, 57), which could have the requisite compliance. Whether the transient development of intimal corrugations has implications for the structure of the hemodynamic boundary layer and fluid mechanical forces on the endothelium remains to be explored. However, the effects of substrate strain on endothelial cells have been extensively investigated (7), demonstrating that they respond to strains of the order of 10% (49). Circumferential strains of this magnitude were measured locally in all vessels during each 10-mmHg increase in transmural pressure. It therefore is probable that changes in the basement membrane, which are characteristic of diseases such as diabetes (1), and endothelial cell integrin expression may affect endothelial cell mechanotransduction (45), as well as the mechanical properties of the intima.

The medial volume is largely occupied by smooth muscle cells. At intermediate pressure (30 mmHg), cell nuclei occupied 20 ± 1% of the medial volume, and since in VSMCs the nuclei comprise 20% of the total cell volume (48) the media is almost completely cellular. This is consistent with the nearly complete absence of fibrillar proteins and would maximize the ability of the vessel to adjust its radius through changes in cellular tone. This ability is further enhanced by the relative stiffness of the adventitia, which forms a stiff boundary for the muscle cells to act against. When the cells are in a passive state the media deforms less than the adventitia under luminal pressure increase, yet has one-tenth the stiffness: luminal pressure is balanced by the circumferential stress in the adventitia, whereas medial compliance allows changes in muscle tone to modulate the inner radius of the vessel. Changes in vascular tone, which have been shown to affect incremental distensibility (47), are likely to lead to a redistribution in circumferential stress, and this will be a target of continuing work.

In most vessels the volume of the adventitia fell as transmural pressure increased, suggesting that as the constraint imposed by the adventitia becomes significant (52) the inner-lying matrix is compressed, as observed in tensile testing (4). These volume changes in the extracellular matrix arise from the exudation of interstitial fluid over the timescale of minutes. This movement of fluid is likely to lead to changes in the interstitial ionic concentration if, as is likely, the matrix has an appreciable fixed charge density. In other tissues such as cartilage these changes are known to influence cellular metabolism (20). Such poroelastic behavior also presents a challenge for the development of models of microvascular wall mechanics incorporating poroelasticity such as those proposed for large vessels (29) and cartilage (33).

In the vessels used in this study the morphology and composition of the adventitia varied more than that of the intima and media, and several biopsies contained vessels with different adventitial morphologies. However, in this small study of subjects of healthy weight, we could establish no correlations between fibrous protein morphology or quantity and variables such as vessel diameter (which varied by a factor of 2) or clinical indexes such as body mass index or blood pressure. Samples from older volunteers may have been stiffened through normal ageing or fibrosis or other undetected pathologies (19). However, a recent study on the mechanics of the adipose tissue (2) from which the vessels were isolated revealed it to be mechanically very heterogeneous, and the adventitial variability may reflect the differing requirements of its role in coupling the vessel to the surrounding tissue. Constrained mixture modeling has been successful in quantifying the mechanical effect of changes in individual fibrous protein networks (8), and similar modeling for microvessels is needed to understand the extent of mechanical variation between the different microvessel morphologies shown in this study.

### 

#### Implications for the understanding of small vessel mechanics and pathology.

Our data could provide the basis of structurally based numerical models of microvessel mechanics. In the meantime it is of interest to discuss them in the context of established models although our work exposes certain limitations. The observation that the media experiences much less circumferential stress than the adventitia cannot be accommodated in a homogeneous model, and as noted above the changes in medial volume during pressurization suggest the need for a poroelastic model. The mechanical model used in this study highlights misconceptions arising from the use of simple homogeneous models but itself has further limitations. It does not take into consideration the effect of internal stress, which is known to be significant in large arteries (5, 15), or the anisotropy, which has been shown morphologically in this work to be significant. There has been extensive work in theoretical modeling of these two factors in large arteries (14, 17, 23, 58), and, to fully understand the mechanical environment of the resistance artery, similar work is needed.

A key target of modeling is to understand the changes associated with hypertension, which is reported to cause vessel walls to become less stiff (26). This behavior has been analyzed in terms of Laplace's equation for cylinder stress, which states that circumferential wall stress increases linearly with lumen radius (41). This equation only applies for a homogenous thin-walled cylinder. In a thick-walled cylinder such as a resistance artery, if homogeneity is assumed the distribution of wall stress is inversely proportional to *r*^2^, placing the peak stress in the cellular intima and media. Decreasing the lumen diameter and increasing the wall-to-lumen ratio reduces the total wall stress, as is considered beneficial, but a greater proportion of the stress is placed on the media, which may be less advantageous. Collagen fiber recruitment has been shown in this study to lead to circumferential stiffening of the adventitia, which causes the vessel wall to thin under increased transmural pressure as the inner layers press up against it. This adventitial stiffening also transfers circumferential stress from the inside of the vessel to the outside. This stiffening effect is commonly misinterpreted as a drop in wall stiffness due to the common practice of quantifying arterial wall mechanics using changes in internal and external diameter. A micromechanical study of diseased vessels is urgently needed to elucidate how the micromechanical environment is changed by pathological vascular remodeling.

#### Limitations.

The statistical power of our analysis was limited by the small number of samples, even though subjects were recruited over a prolonged period. A primary factor was that, of the abdominal subcutaneous fat biopsies, only 70% yielded a suitable resistance artery, and, of the 12 arteries obtained, only 7 were suitable for mechanical analysis. It may be that other sampling sites would be more productive. Articles citing buttock biopsies as the source of subcutaneous tissue do not report such problems (43). Because of the small sample numbers, we were unable systematically to vary smooth muscle tone, which is known to make a variable contribution to mechanics (22). Instead, the vessels were imaged at what we presumed to be basal tone, giving pressure-diameter curves in the middle of their expected range (47).

Another mechanical parameter that was poorly controlled was longitudinal tension. What longitudinal tensions a microvessel might experience in adipose tissue in vivo is an interesting question, and in the absence of an answer we mounted vessels at the minimal straightened length. Changing luminal pressure altered longitudinal strain, and, by tracking fiducial markers on the adventitial surface of a single vessel, we found this to be, on average, 2.9% over the pressure range employed. This is small compared with the radial distension but could be examined more rigorously using digital image correlation techniques, and it may be important to incorporate such information in finite element models. Another uncertainty was the physiological pressures in the microvessels. It may have been higher than the range 3–50 mmHg we employed, but this was chosen as being the one over which most structural changes occurred: extending the range would have meant unacceptable extension of the imaging time.

#### Conclusion.

The three fibrous protein networks in human subcutaneous resistance arteries have been imaged at incremental transmural pressures in three dimensions at high resolution, and they have been found each to possess unique mechanical characteristics. A two-layer mechanical model predicts that the adventitia is significantly stiffer than the media at pressures sufficient to recruit its extensive network of collagen and therefore bears the vast majority of the circumferential stress in the passive state. Orientation analysis provides a first step toward understanding the anisotropic nature of the vessel wall. Our findings have implications for the understanding of small artery biomechanics and related pathologies.

## GRANTS

The research was funded by British Heart Foundation Grant No. PG/11/17/28788 and by the National Institute for Health Research (NIHR) Exeter Clinical Research Facility.

## DISCLOSURES

The views expressed are those of the author(s) and not necessarily those of the NHS, the NIHR, or the Department of Health. No conflicts of interest, financial or otherwise, are declared by the author(s).

## AUTHOR CONTRIBUTIONS

J.S.B., A.A., A.P., and L.H. performed experiments; J.S.B. analyzed data; J.S.B., C.E.T., A.C.S., J.W., and C.P.W. interpreted results of experiments; J.S.B. prepared figures; J.S.B. drafted manuscript; J.S.B., A.A., C.E.T., A.C.S., J.W., and C.P.W. edited and revised manuscript; J.S.B., A.A., A.P., L.H., C.E.T., A.C.S., J.W., and C.P.W. approved final version of manuscript; A.C .S., J.W., and C.P.W. conceived of and designed research.

## Supplementary Material

Supplemental Video 1A

Supplemental Video 1B

Supplemental Video 1C

Supplemental Video 1D

Supplemental Video 2A

Supplemental Video 2B

Supplemental Video 2C

Supplemental Video 2D
